# Effect of androgen deprivation therapy on bone mineral density in patients with prostate cancer

**DOI:** 10.12669/pjms.38.5.5446

**Published:** 2022

**Authors:** Kashaf Ilyas, Zainab Hafeez, Rukhsana Latif

**Affiliations:** 1 Dr. Kashaf Ilyas, MBBS, Department of Surgery, Nishtar Medical University & Hospital, Multan, Pakistan; 2 Dr. Zainab Hafeez, MBBS, Department of Surgery, Nishtar Medical University & Hospital, Multan, Pakistan; 3 Dr. Rukhsana Latif, MBBS, Department of Surgery, Nishtar Medical University & Hospital, Multan, Pakistan

**Keywords:** Bone mineral density, prostate cancer, androgen deprivation therapy, bone metabolism markers

## Abstract

**Objectives:**

To access the effects of acute and chronic Androgen Deprivation Therapy on Bone Marrow Density and related bone markers; to compare the bone loss among the patients who terminated GnRH use and control group (not given ADP therapy at all) with the ones with acute or chronic Androgen Deprivation Therapy.

**Methods:**

A cross-sectional study was conducted in the Oncology Department of Nishtar Medical University & Hospital Multan for one year. Bone mineral density of the entire body, 1/3^rd^ distal radius, ultra-distal forearm, femoral neck, and lumbar spine, was measured in 40 patients diagnosed with non-metastatic prostate cancer at baseline for the duration of six months. They were categorized into four groups: (i) acute ADT (less than six months of treatment; (ii) chronic ADT (greater than six months of treatment; (iii) former ADT; and (iv) no ADT (placebo groups). Quantitative measures of bone metabolism marker, including C-terminal cross-linking telopeptide of type I collagen (CTX) and procollagen type I N-terminal propeptide (PINP) was done.

**Results:**

The cross-sectional analysis showed that BMD dropped significantly in more in patients with former ADT or control groups as compared to chronic ADT. At the 6^th^ month assessment, a significant decline in ultra-distal forearm BMD was demonstrated in patients from both acute and chronic ADT groups (4.05% and 2.54%, *P* = .001 and .016, respectively). Total body BMD was significantly reduced among those on acute treatment (2.91%, p=0.022). In the former ADT group, a significant increase of BMD was observed in the femoral neck and lumbar spine bones (1.60 % and 2.85%, *P* = .001 and .0064, respectively). The difference of changes in BMD of the acute and chronic groups was not significant. The levels of PINP and CTX levels were significantly increased in an chronic and acute group than in placebo or former ADT groups.

**Conclusion:**

Chronic and acute ADT users experience similar changes in BMD levels but reversibility of BMD can be achieved on withdrawal of treatment. Similarly disturbed bone metabolism markers come back in range on withdrawal of treatment.

## INTRODUCTION

Androgen deprivation therapy is the established therapeutic approach for metastatic prostate cancer (PCa), which is acquired either through administering gonadotrophin-releasing hormone (GnRH) agonist (medical castration) or through surgical intervention (bilateral orchiotomy), with or without antiandrogens.^1^ Similarly, ADT is also coupled with radiotherapy for the management of non-metastatic but locally advanced or high risk prostate cancers.^2^

Undertaking ADT, however, may produce harmful effects on bone health such that bone density is rapidly reduced, enhancing the risk of bone fractures.^3^ Majority of the related studies in the literature are cross-sectional in nature and reported the significant decrease in Bone mineral density (BMD) in patients who received ADT than those who didn’t receive it or the healthy individuals.^4^,^5^ Only few longitudinal studies have been carried out so far to evaluate the association between ADT and bone loss. Some studies demonstrated a higher bone less in first year of ADT use than the subsequent year.^6^ However, a contrasting longitudinal study, conducted on recently diagnosed advanced PCa cases, showed BMD kept on declining for seven consecutive years independent of the baseline BMD levels with the GnGH agonist treatment, despite the regular administration of vitamin D and calcium supplements.^7^ This predict the inefficacy of commonly used vitamin D and calcium supplements in preventing ADT-related bone loss. In another similar study, authors analyzed 65 patients with non-metastatic PCa, 35 of them were already having GnRH treatment before the start of the study while 42 started taking the treatment at the onset of the study. The study concluded that the hip BMD declined regardless the ADP status of the patients, new users or those with the previous history of the treatment.^8^ Another study evaluated the BMD status following the termination of the GnRH agonist treatment and found that even 1 year after the discontinuation of the treatment, BMD decreased due to persistent suppression of testosterone.^9^

In recent years, it has been found that intermittent ADT help-s in testosterone recovery and which improves the ADT-induced bone loss.^10^,^11^ The present study was designed to access the effects of acute and chronic ADT on BMD and related bone markers. It was also aimed to compare the bone loss among the patients who terminated GnRH use and control group (not given ADP therapy at all) with the ones with acute or chronic ADT.

## METHODS

A cross-sectional study was conducted at Urology and Oncology Department of Nishtar Medical University & Hospital Multan for the period of one year from 6^th^ July 2020 to 6^th^ July 2021. A total of 40 men, aged 50 or above and diagnosed non-metastatic prostate cancer were included in the study. The patients had variable history and current status of undergoing treatment such as ADT (for different treatment spans) with or without radical prostatectomy, radian therapy or active surveillance. The participants diagnosed with renal failure, severe hepatic disorder, hyperthyroidism, hyperparathyroidism, rheumatoid arthritis, Paget’s disease or administered with such medication that affect bone health were excluded from the study to avoid the confounding effects on study’s results. Informed consent was sought from all the included participants and approval was taken from ethical review committee of the hospital.(Ref.121-50-NMU June 19, 2020)

All the patients had dual-energy x-ray absorptiometry (DXA) scans and blood samples in fasting state were collected prior to and following the x-ray. Soon after the blood sample collection, their serum was separated. The participants of the study were grouped into four categories: 1) patients who underwent GnRH agonists therapy for 6 months and less; 2) patients who had been treated with GnRH agonists for more than 6 months at the time of study; 3) patients who had previously been administered GnRH agonists but later terminated the treatment or patients who were at intermittent ADT at study initiation time, and 4) patients who had never been administered ADP. Baseline clinical history such as prostate specific antigen at diagnosis, biopsy results, and tumor stage was obtained from hospital records. Participants were then individually asked about therapeutic management history and the time span of each treatment (the data was compared with hospital records). The participants were also asked about the use of calcium or vitamins D supplements. BMD of total body, 1/3^rd^ distal radius, ultra distal forearm, femoral neck, L2-L4 lumbar spine was measured at the start of the study and at 6^th^ month by DXA scans. T-score of evaluated regions was used to classify patients: normal (> 1SD); osteopenic (between -1 to -2.5), osteoporotic (≤ -2.5), as per World Health Organization criteria. Automatic immunoassay were run to measure the serum levels of 25-hydroxyvitamin D, PTH, estradiol (E2), luteinizing hormone (LH), follicle-stimulating hormone (FSH), and Testosterone and serum levels of bone metabolism marker, including C-terminal cross-linking telopeptide of type I collagen (CTX) and procollagen type I N-terminal propeptide (PINP) were also measured.

SPSS (version 18.0) was used for statistical analysis. Baseline BMD and sex steroid hormones levels are compared between four study groups Continuous variables were presented in the form of median and range and comparison between the study groups was done through Kruski-Wallis test. If the difference turned out to be significant, Wilcoxon test was performed to conduct pairwise comparison. Categorical data was expressed as numbers and percentages and comparison was made through Fisher exact test. Difference in proportions of normal BMD, osteopenia, and osteoporosis between study groups was compared by proportion test. Least square (LS) mean values along with standard error (SE) were computed to determine % difference in baseline levels of BMD till six months. Intra-group change in BMD was assessed through student’s t-test. A P-value less than 0.05 was considered statistically significant.

## RESULTS

The baseline characteristics of patients in all study groups are presented in Table-I. No significant difference was found between 4 study groups in terms of demographics. Majority of patients in each group: 4 (57.5%) patients in acute ADT group had GnRH agonist monotherapy; 5 (55.5%) patients in chronic ADT group had GnRH agonist monotherapy and another 55.5% had radiation therapy; 8 (57.1%) patients had GnRH agonist monotherapy, and 6 (60%) in control group had radiation therapy along with surgery (Table-I). Similarly, no significant difference was found in terms of clinical staging of the cancer, calcium and vitamin D intake, and prior fracture incidence However, the PSA was significantly higher in ADT undergone groups than control group (p<0.01) (Table-II). Difference in baseline level of BMD between four study groups is shown in Table-III. Total body BMD of was significantly lesser in chronic ADT users than former ADT and control groups (median 1.0967 g/cm2 vs 1.183 and 1.215, respectively). However, no significant difference in BMD was found at the lumber spine, 1/3^rd^ distal radius, ultra-distal forearm, and femoral neck (p>0.05). The analysis of worst site of BMD at baseline level revealed, 28.5% and 42.8% incidence rate of osteopenia and osteoporosis, respectively, in acute ADT group; 66.6% and 11.1%, respectively, in chronic ADT group; 64.2% and 14.2%, respectively, in former ADT users, and 10% incidence rate of both disorders in control group (Table-IV). Significant difference was found in baseline level of evaluated sex hormones, including FSH, LH, estradiol, and testosterone, across the 4 study groups (all P < .05) (Table-V). Mean baseline level of testosterone hormone was significantly lesser in acute ADT users and chronic ADT users than in former ADT and control groups (.047 and .067 ng/mL vs 3.74 and 4.33 ng/mL, respectively). Similarly, mean levels of estradiol, LH, and FSH were significantly lesser in acute and chronic group as compared to formal ADT and control groups. However, no significant difference was found in Serum 25-hydroxyvitamin D and PTH levels across 4 group (Table-V). Baseline serum PINP and CTX levels, however, were significantly raised in adult and chronic ADT group than in control group (PINP: 75.68 and 70.62 ng/ mL vs 48.46 ng/mL; CTX: 0.73 or 0.61ng/mL 0.47 ng/mL, respectively).

**Table I:** Baseline Characteristics of Study Groups (N=40).

Variables	Acute ADT	Chronic ADT	Former ADT	PCa Control	P-value
Total participants	7.0	9	14	10	
Age (years)	70.1(58–79)	69.5 (55–82.3)	73.40 (52.9–81.6)	63.70 (57.7–81.5)	0.64
Weight (Kg)	86.1 (75.7–105.0)	83.2 (68.9–112)	75.30 (64.8–116.6)	82.25 (55.5–123.4)	0.34
Height (cm)	171 (165.5–178.0)	173.3 (159–192)	172.20 (157.5–183)	176.50 (157.5–186)	0.45
BMI	31.50 (25.2–37.8)	30.30 (21.4–42.0)	25 (20.3–37.0)	25.65 (18.20–34.8)	0.085
**Type of ADT, n (%)**					
GnRH agonist monotherapy	4 (57.1%)	5 (55.5%)	8 (57.1%)	0 (0)	
GnRH agonist with antiandrogen	3 (42.8%)	4 (44.4%)	6 (42.8%)	0 (0)	
Radiation therapy	2 (28.5%)	5 (55.5%)	11 (78.5%)	7 (70%)	
Surgery	0 (0)	1 (11.1%)	1 (7.1%)	4 (40%)	
Radiation therapy and surgery	1 (14.2%)	2 (22.2%)	3 (21.4%)	6 (60%)	
Active surveillance	0 (0)	0 (0)	0 (0)	4 (40%)	

**Table II:** Clinical and Medication Factors of Study Groups (N=40).

Variables	ADT and former ADT groups (N = 30)	PCa controls (N = 10)	P-value
PSA, median (range)	12.55 (1.50–14.5)	6.60 (1.9–10.4)	<0.01
**Clinical stage (n, %)**			0.054
T1	6 (20%)	1 (10%)	
T2	7 (23.3%)	5 (50%)	
T3	10 (33.3%)	1 (10%)	
T4	1 (3.33%)	0 (0)	
Unknown	4 (13.3%)	3 (30%)	
**Baseline calcium use (n, %)**			.64
Yes	5 (16.6%)	3 (30%)	
No	25 (83.3%)	7 (70%)	
**Baseline vitamin use (n, %)**			.83
Yes	7 (23.3%)	3 (30%)	
No	23 (76.6%)	7 (70%)	
**Prior fragility fracture**	5 (16.6%)	2 (20%)	.88

**Table III:** Baseline BMD of the of Study Groups (N=40).

	Acute ADT (n=7)	Chronic ADT (n=9)	Former ADT (n=14)	PCa Control (n=10)	P-value
L2-L4 (g/cm2)	1.284 (1.057–1.398)	1.185 (.619–1.643)	1.393 (.914–1.719)	1.327 (.890–1.785)	.091
Femoral neck (g/cm2)	0.928 (0.593–1.099)	.871 (.708–1.282)	0.882 (.720–1.296)	0.946 (.744–1.217)	.34
1/3^rd^ distal radius (g/cm2)	.928 (.644–1.105)	.919 (.581–.123)	0.949 (.774–1.168)	0.989 (.722–1.217)	.076
Ultradistal forearm (g/cm2)	.479 (.350–0.543)	.445 (.335–.605)	0.478 (.388–0.670)	0.485 (.340–0.696)	.2
Total body (g/cm2)	1.174 (.971–1.476)	1.097 (.919–1.097)	1.183 (.962–1.189)	1.215 (.880–1.463)	<0.05

**Table IV:** Frequency of Normal BMD, Osteopenia, Osteoporosis among the Study Groups (N=40).

	Acute ADT (n=7)	Chronic ADT (n=9)	Former ADT (n=14)	PCa Control (n=10)
**Lumbar spine (n, %)**				
Normal	7 (100%)	6 (66.6%)	10 (17.4%)	8 (80%)
Osteopenia	0	2 (22.2%)	3 (21.4%)	1 (10%)
Osteoporosis	0	1 (11.1%)	1 (7.1%)	1 (10%)
**Femoral neck**				
Normal	2 (28.5%)	3 (33.3%)	4 (28.5%)	7 (70%)
Osteopenia	3 (42.8%)	5 (55.5%)	8 (57.1%)	2 (20%)
Osteoporosis	2 (28.5%)	1 (11.1%)	2 (14.2 %)	1 (10%)
**Site with worst BMD**				
Normal	2 (28.5%)	2 (22.2%)	3 (21.4%)	8 (80%)
Osteopenia	2 (28.5%)	6 (66.6%)	9 (64.2%)	1 (10%)
Osteoporosis	3 (42.8%)	1 (11.1%)	2 (14.2%)	1 (10%)

**Table V:** Baseline levels of Serum Hormones (N=50).

	Acute ADT (n=7)	Chronic ADT (n=9)	Former ADT (n=14)	PCa Control (n=10)	P-value
Testosterone (ng/mL)	.047 (.026–3.31)	.061 (.024–.914)	3.741 (0.026–8.335)	4.331 (.046–7.310)	<0.05
LH (mIU/mL)	.105 (.105–.255)	.103 (0.100–.670)	10.535 (0.100–40.582)	7.29 (.16–23.5)	<0.05
FSH (mIU/mL)	5.51 (.96–12.11)	4.27 (1.62–12.65)	17.85 (2.52–53.68)	9.66 (3.34–51.57)	<0.05
Estradiol (pg/mL)	5.1 (5.1–108.68)	5.0 (5.0–27.54)	24.43 (6.00–43.98)	23.26 (5.05–38.45)	<0.05
Parathyroid hormone (pg/mL)	38.24 (33.17–65.85)	41.97 (17.46–86.68)	42.22 (22.68–76.15)	40.91 (23.62–86.83)	0.92
25-Hydroxyvitamin D (ng/mL)	24.15 (18.62–33.90)	30.91 (14.27–65.50)	30.99 (14.04–58.84)	31.55 (11.57–52.68)	0.54
PINP, ng/mL	75.68 (67.21–110.50)	70.62 (41.22–136.61)	46.17 (30.35–125.82)	48.49 (27.13–106.8)	0.001
CTX, ng/mL	.73 (.41–1.30)	.61 (.21–1.24)	.376 (.188–1.189)	.471 (.259–.975)	0.003

**Fig.1:**
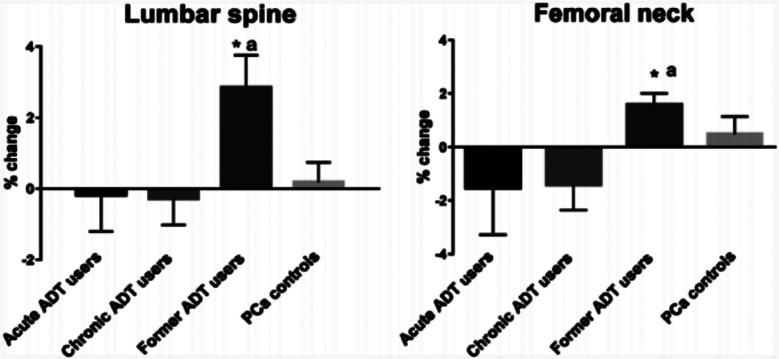
LS mean percentage change (with SE) from baseline BMD Levels at lumbar spine and femoral neck

**Fig.2:**
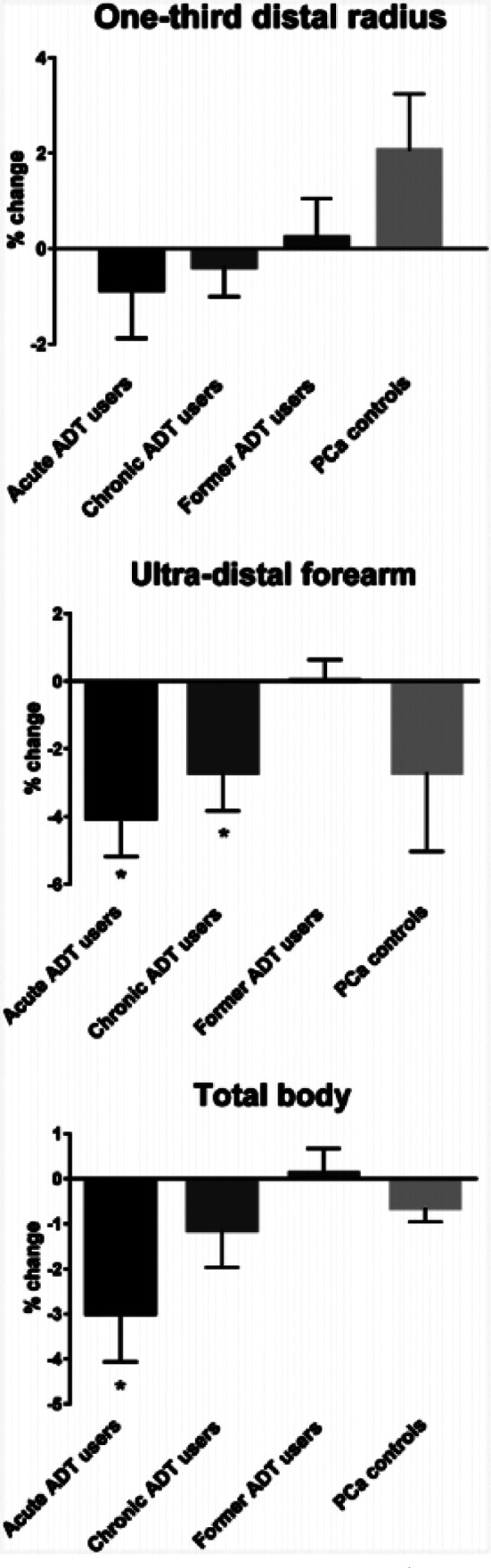
LS mean % change (with SE) in baseline BMD at 1/3^rd^ distal radius, ultra-distal forearm, and total body BMD.

**Fig.3:**
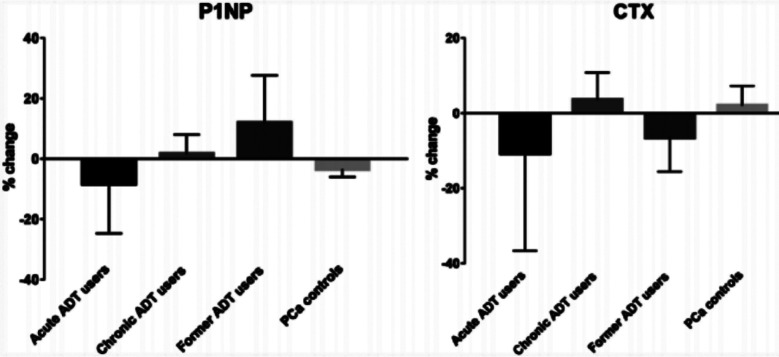
LS mean % change in baseline levels of serum PINP and CTX.

Following six months follow up period, former ADT group demonstrated significant increase in BMD in lumber spine (+2.85%, P = .0064). Whereas, no significant changes in baseline BMD levels was found in lumbar spine of acute ADT (−0.16%, P = .97), chronic ADT (−0.21%, P = .52), and control (+0.19%, P = .34) groups. The 6-month followed-up levels of BMD at lumber spine were significantly different across four groups (p<0.05) (Fig.1). Similarly, formal ADT group reported significant increase in BMD in femoral neck (+1.60%, P = .001), as compared to acute users (−1.51%, P = .41), chronic ADT group (−1.41%, P = 0.20), and control group (+0.50%, P = .52). The change in femoral neck BMD was significant in former ADT users compared to other groups (Fig.1). In case of 1/3^rd^ distal radius, BMD was increased in control group; however the change remained non-significant (+2.12%, P = .094). Similarly, no significant change in levels was found in other groups and hence difference in changed BMD level across four groups remained in significant (Fig.2). Patients in acute and chronic ADT reported significant decline in ultradistal forearm BMD from baseline levels (−4.05%, P = .001and 2.54%, P = .016, respectively), as compared to other groups who reported no significant change (Fig.2). Total BMD was significantly reduced in acute ADT users (−2.91%, P = .022), than non-significant changes in controls (−0.613%, P = .060), chronic ADT group (−1.25%, P = .12) and former ADT group (+0.15%, P = .91) (Fig.2). Fig.3 presents LS mean % changes in serum CTX and PINP. The change in baseline levels of these bone markers was not significant across 4 groups. The LS mean changes in PINP values among acute ADT group, chronic ADT group, former ADT group, and PCa controls group were −8.31%, +1.52%, +11.93%, and −3.41%, respectively. Whereas for serum CTX,, LS mean baseline changes were −10.73%, +3.55%, −6.52%, and +2.17%, for the corresponding groups respectively. (Fig.3).

## DISCUSSION

The study reported reduced total body BMD levels in men undergoing long term ADT than former ADT users or those with had never gone through this treatment. These study results are in compliance with previous related cross-sectional studies.^4^,^12^ Additionally, it was also noted that former ADT user’s experienced significant increase in BMD levels which predicted the probability of reversibility in ADT-associated bone loss. The study also revealed incidence of bone disorders (osteoporosis) was lower in cancer suffering men who didn’t undergo ADT but the difference was not significant than other study groups. Similar results were found by earlier studies which compared osteoporosis prevalence rate between ADT users and on-users.^13^ In a study conducted by Moorte et al ADT users and non-users were compared for incidence of osteoporosis for 6 years and thus, the significant difference was found between two groups.^14^ Thus, shorter duration of our study might be a factor behind non-significant association between ADT and osteoporosis.

In the present study, 80% of patients within control group had normal BMD against 20% men in chronic ADT group. Similar results were reported by Morote et al who found larger number of patients with normal BMD in control group than among ADT users (28.1% vs 13.2%, P = .035). The six months follow-up in this study showed that acute ADT users witnessed decline in BMD within range from 0.16% to 4%. However, decline in total body and ultra-distal BMD values from baseline was found to be significant. Similarly, Mittan et al, reported maximum decline in ultra-distal BMD (5.3%) among ADT users.^15^ The literature suggests that forearm BMD strongly predicts incidence of osteoporotic fractures in male population^16^ and this study confirms forearm as a preferred site for BMD assessment. The study reported non-significant difference of BMD changed levels across four study groups. Contrastingly, 12-month long study Acute users showed significant BMD reduction than other evaluated groups.^17^ The difference can be justified from shorter duration and limited sample size of our study. The recovery of lumber spine and femoral neck BMD in former ADT users of our study was surprising. In a study conducted by Yu et al, BMD changes were found among the patients on intermittent therapy. The study reported maximum improvement in lumber spine BMD levels during first off treatment period.^18^

This study showed significant higher levels bone formation and resorption markers (PINP and CTX, respectively) in acute and chronic users than other study groups, indicating increase bone turn over in patients who are actively treated with ADT. Varsavsky et al, conducted a similar study and found increased levels bone formation (BSAP) and bone resorption (CTX) in patients undergoing active ADT treatment.^19^ Whereas, Greenspan et al, reported significant rise in PINP levels in acute ADT users but not in chronic ADT users as compared to non-ADT users. The authors identified PINP as a distinguishing marker between acute and chronic PINP users.^17^ The study found no significant difference in bone markers over six month period across four groups which again could be due to smaller sample size and shorter follow up period, the major limitations of study. Moreover, the patients were not randomized to receive desired treatment which could have produced bias in the results.

## CONCLUSION

Acute and chronic ADT users experience similar changes in BMD levels but reversibility of BMD can be achieved on withdrawal of treatment. Similarly, disturbed bone metabolism markers come back in range on withdrawal of treatment.

### Authors Contribution:

**KI, ZH:** Conceived, designed and did statistical analysis & editing of manuscript.

**RL, ZH:** Did data collection and manuscript writing.

**ZH, KI:** Did review and final approval of manuscript.
